# Framework for the quantitative assessment of adaptive radiation therapy protocols

**DOI:** 10.1002/acm2.12437

**Published:** 2018-08-29

**Authors:** Sarah Weppler, Harvey Quon, Robyn Banerjee, Colleen Schinkel, Wendy Smith

**Affiliations:** ^1^ Department of Physics and Astronomy University of Calgary Calgary AB Canada; ^2^ Department of Medical Physics Tom Baker Cancer Centre Calgary AB Canada; ^3^ Department of Radiation Oncology Tom Baker Cancer Centre Calgary AB Canada; ^4^ Department of Oncology University of Calgary Calgary AB Canada

**Keywords:** adaptive radiation therapy, quality assurance, head and neck cancer

## Abstract

**Background:**

Adaptive radiation therapy (ART) “flags,” such as change in external body contour or relative weight loss, are widely used to identify which head and neck cancer (HNC) patients may benefit from replanned treatment. Despite the popularity of ART, few published quantitative approaches verify the accuracy of replan candidate identification, especially with regards to the simple flagging approaches that are considered current standard of practice. We propose a quantitative evaluation framework, demonstrated through the assessment of a single institution's clinical ART flag: change in body contour exceeding 1.5 cm.

**Methods:**

Ground truth replan criteria were established by surveying HNC radiation oncologists. Patient‐specific dose deviations were approximated by using weekly acquired CBCT images to deform copies of the CT simulation, yielding during treatment “synthetic CTs.” The original plan reapplied to the synthetic CTs estimated interfractional dose deposition and truth table analysis compared ground truth flagging with the clinical ART metric. This process was demonstrated by assessing flagged fractions for 15 HNC patients whose body contour changed by >1.5 cm at some point in their treatment.

**Results:**

Survey results indicated that geometric shifts of high‐dose volumes relative to image‐guided radiation therapy alignment of bony anatomy were of most interest to HNC physicians. This evaluation framework successfully identified a fundamental discrepancy between the “truth” criteria and the body contour flagging protocol selected to identify changes in central axis dose. The body contour flag had poor sensitivity to survey‐derived major violation criteria (0%–28%). The sensitivity of a random sample for comparable violation/flagging frequencies was 27%.

**Conclusions:**

These results indicate that centers should establish ground truth replan criteria to assess current standard of practice ART protocols. In addition, more effective replan flags may be tested and identified according to the proposed framework. Such improvements in ART flagging may contribute to better clinical resource allocation and patient outcome.

## 1 INTRODUCTION

Due to the close proximity of target volumes to organs at risk (OAR) in patients with head and neck cancer (HNC), intensity‐modulated radiation therapy (IMRT) or volumetric‐modulated arc therapy (VMAT) techniques are commonly used. The steep dose gradients characteristic of these conformal external beam methods allow better target coverage and OAR sparing compared to less conformal external beam techniques; however, setup uncertainties and anatomical changes limit adherence to planned dose deposition throughout treatment fractionation.

Adaptive radiation therapy (ART) protocols replan treatment in response to anatomical changes to ensure that planned target coverage and OAR sparing are achieved. While successful ART approaches may improve clinical outcomes,^1^, ^2^, ^3^ they are resource intensive.^4^, ^5^ Therefore, the clinical viability of ART depends on correctly identifying patients most likely to benefit from a replanned treatment. Selection criteria in the literature generally fall into three categories. Image‐based methods compare periodic cone beam CT (CBCT) or CT images with the CT simulation (CTsim) to identify any systematic physical changes.^6^, ^7^, ^8^, ^9^, ^10^ Temporally based methods preselect the time at which a new plan should be calculated.^2^, ^11^ Patient characteristic‐based methods examine pretreatment parameters such as weight and tumor stage to predict if and when a replan may be necessary.^8^, ^12^ In most protocols, these parameters indicate when a physician should make a judgment call regarding possible adjustment to immobilization, re‐CT, dose recalculation, or replanning. Few dosimetric thresholds warranting a replan have been stated and efficient and easily implementable replan flags remain elusive.^2^, ^3^, ^8^, ^13^, ^14^

Despite the variety of ART protocols used clinically, the accuracy of simple standard of practice ART replan candidate identification is rarely quantified in the literature. This work proposes a two‐step, quantitative evaluation framework and exhibits its utility through the assessment of a single institution's clinical ART flag: a change in body contour exceeding 1.5 cm. Anecdotally, this type of flag is commonly used in many institutions. First, “ground truth” for dosimetric deviations requiring replanning were established by surveying radiation oncologists (ROs) treating HNCs. Second, flag performance was quantitatively assessed by comparing this “truth” to interfractional dose deviations. In this study, we assessed 15 HNC patients treated with VMAT whose body contour changed by >1.5 cm at some point in this treatment. This method of quantifying ART performance may allow clinics to identify more effective flags to improve clinical resource allocation and ultimately patient outcomes.

## 2 METHODS

### 2.A Protocol

For HNC VMAT patients treated in this study, kV‐CBCT images were acquired approximately every five fractions. For select cases, CBCTs were also acquired for the first three fractions to assess setup reproducibility; CBCT acquisition was delayed until a later fraction if the patient was feeling unwell, due to the prolonged on‐unit time, and CBCT images may have been taken on the day after a flag in body contour change for additional monitoring. Patients were imaged on the treatment couch using CBCT after kV‐orthogonal x‐ray acquisition and subsequent couch position adjustment and prior to treatment delivery. Radiation therapists performed a rigid registration of each CBCT with the CTsim according to institutional image‐guided radiation therapy practices. The axial view of the rigid registration was then assessed to identify, for any axial slice, the largest pointwise distance between the CBCT and CTsim external contours. The latter was used to quantify change in body contour and formally may be regarded as a maximum axial slice‐based Hausdorff distance. In practice, this flagged weight loss and tumor shrinkage effects as well as changes in shoulder position. Those patients exhibiting a change in body contour exceeding 1.5 cm were “flagged” for consult with a medical physicist. The RO in collaboration with the physicist would then elect to refit the immobilization, re‐CT, and/or replan treatment; clinicians may have elected to monitor patients if only a few (e.g., less than 5) fractions remained.

### 2.B Patients

Fifteen consecutively flagged patients exhibiting a greater than 1.5 cm change in body contour were retrospectively enrolled in this study. Inclusion criteria were: patient age greater than 18 years; treated with volumetric‐modulated arc therapy (VMAT); completion of a radical/curative 70 Gy in 33 fraction dose regimen; forwarded at least once for physicist consult between December 2015 and March 2016 after radiation therapist identification of a >1.5 cm change in external body contour.

The study was identified as a minimal‐risk quality improvement investigation according to the institutional research ethics board (Alberta Innovates Health Solutions and A pRoject Ethics Community Consensus Initiative). Further review by a research ethics board was not required according to institutional mandate.

### 2.C Radiation treatment planning

Patients were immobilized with Aquaplast RT split‐frame U‐shaped head masks (Qfix, Avondale, PA, USA) with Instaform™ two‐part foam shoulder supports (CDR Systems, Calgary, AB, Canada). CTsim images were acquired for all patients, in addition to PET and MR images as needed for target delineation. Gross tumor volumes (GTV) were identified by the treating RO via the acquired images, nasopharyngoscopy, and palpation. Clinical target volumes (CTV) were obtained by 5–10 mm extension of the GTV; planning target volumes (PTV) were obtained by a 3 mm margin on the CTV. Planning organ at risk volumes (PRV) for the brainstem and spinal cord were created with 3 and 5 mm structure margins, respectively. 70 Gy in 33 fractions (2.12 Gy/fraction) was prescribed to the high‐risk PTV with 59.4 Gy (1.8 Gy/fraction) to the low‐risk PTV, including prophylactic nodal coverage.

VMAT plans were calculated in the Eclipse™ Treatment Planning System, Version 11 (Varian Medical Systems, Palo Alto, CA, USA) using the Anisotropic Analytical Algorithm (AAA) convolution–superposition dose calculation model.

### 2.D Protocol assessment

A survey was circulated to HNC ROs at our center and others in Canada; ROs were asked to provide a percentage violation of the planning objective, as observed for a single assessed treatment fraction, that they would consider warranted a treatment replan. Median responses of the five completed surveys formed the basis of the proposed “major/minor violation” replan criteria and served as ground truth in protocol evaluation.

Dosimetric parameters were calculated for each treatment fraction with CBCT acquisition (106 CBCT images total for 15 patients). The CTsim image was deformed to a given CBCT image (SmartAdapt^®^, Varian Medical Systems, Palo Alto, CA, USA). Contours were propagated to the deformed CTsim and corrected as needed by the first author under the guidance of an experienced dosimetrist. The result yielded a contoured “synthetic CT.” The originally planned beam angles, multileaf collimator apertures, and monitor units were copied and realigned to the deformed CTsim; realignment was performed manually in reference to rigid registrations and external markers as DICOM links between images had to be removed prior to deformable image registration. Dose deposition for each synthetic CT was recalculated in Eclipse. The assigned HU values for dental artifact correction structures reverted to image values during this process. A subset of five patients with varying degrees of dental correction were assessed; dose parameter values relevant to this study varied by at most 0.2 Gy over the course of treatment and this known discrepancy was concluded to have a minimal effect on truth‐table analysis results.

Dose accumulation was calculated by linear interpolation of dose parameter values for fractions without CBCT acquisition. Dose warping capabilities, which may have improved the accuracy of dose accumulation estimates, were not available at our center at the time of this analysis. Therefore, parameter values were averaged over 33 fractions and gave a conservative estimate of overall dose deposition by assuming, for example, that Dmax occurred in the same spatial location. Truth tables were used to calculate the sensitivity, negative predictive value, etc., of the protocol for all major and/or minor violations, OAR, and target volumes.

It should be noted that while this analysis is biased by the fact that only flagged patients are included in the study cohort, the resulting sensitivity provides a theoretical upper bound for that of a cohort comprised of both flagged and unflagged individuals. Limited sensitivity observed for the present cohort implies a limited sensitivity persists in general (Appendix A).

## 3 RESULTS

The December 2015–March 2016 accrual of the 15 patient cohort (Table 1) corresponded to 35 individual flaggings as many of these patients exhibited >1.5 cm change in body contour at multiple times during treatment. This is approximately 1/3 of the estimated 48 HNC patients treated with curative intent radiation therapy during this time.

**Table 1:** Characteristics of the patient cohort: 15 patients exhibiting a change in external body contour >1.5 cm

Patient	Primary site	Subsite	Local stage	Nodal stage	Stage	Chemotherapy	HPV status (p16 status)
1	Hypopharynx	Right pyriform sinus	T2	N3	IVB	Cisplatin	Unknown
2	Oropharynx	Left tonsil	T3	N2b	IVA	Cetuximab	Positive
3	Oropharynx	Right tonsil	T2	N2b	IVA	Cisplatin	Positive
4	Unknown primary		TX	N2b	IVA	Cisplatin	Positive
5	Oropharynx	Right base of tongue	T4a	N2c	IVA	Cetuximab	Positive
6	Oropharynx	Right base of tongue	T4a	N2a	IVA	Cisplatin	Positive
7	Nasopharynx		T3	N2	III	Cisplatin	Negative
8	Unknown primary		TX	N2c	IVA	Cisplatin	Positive
9	Oropharynx	Left base of tongue	T4a	N2c	IVA	Cetuximab	Positive
10	Oropharynx	Right base of tongue	T3	N2b	IVA	Cisplatin	Positive
11	Nasopharynx		T2	N2	III	None	Unknown
12	Nasopharynx		T1	N1	II	Cisplatin	Unknown
13	Oropharynx	Right base of tongue	T3	N2c	IVA	Cisplatin	Positive
14	Nasopharynx		T4	N1	IVA	Cisplatin	Unknown
15	Oropharynx	Right tonsil	T4a	N2c	IVA	Cisplatin	Positive

### 3.A Survey results

Survey results are shown in Table 2. Values are stated with respect to institutional planning objectives or initial plan parameter values in the absence of formal planning criteria. Structure‐specific unacceptable violations provided by the ROs were subsequently stratified into “major violations” and “minor violations” based on the magnitude of median responses and the relevance to treatment outcome, for example, target coverage and brainstem/spinal cord sparing vs target hot spot and parotid sparing, respectively. Optic structure and parotid gland limits were not originally included in the survey, but were each added with proposed unacceptable violations by a RO and so included in Table 2. Optic structures were considered major violation criteria, but no violations were observed in this patient cohort. Unacceptable violations for GTV and CTV structures were inferred from PTV violations as no formal planning constraints are quantified for these structures; therefore, no ranges are provided for these values in the table. The number of fractions and number of patients with each violation were also recorded. Unacceptable violations of planned parameter values largely occurred in the PTV volumes and parotid glands, with a similar proportion of violations persisting in the dose accumulation. The median values of RO survey responses (percentage unacceptable violation of the planning criteria) were used as truth for this study: patient treatment fractions should have been flagged if they exhibited one or more unacceptable violations.

**Table 2:**
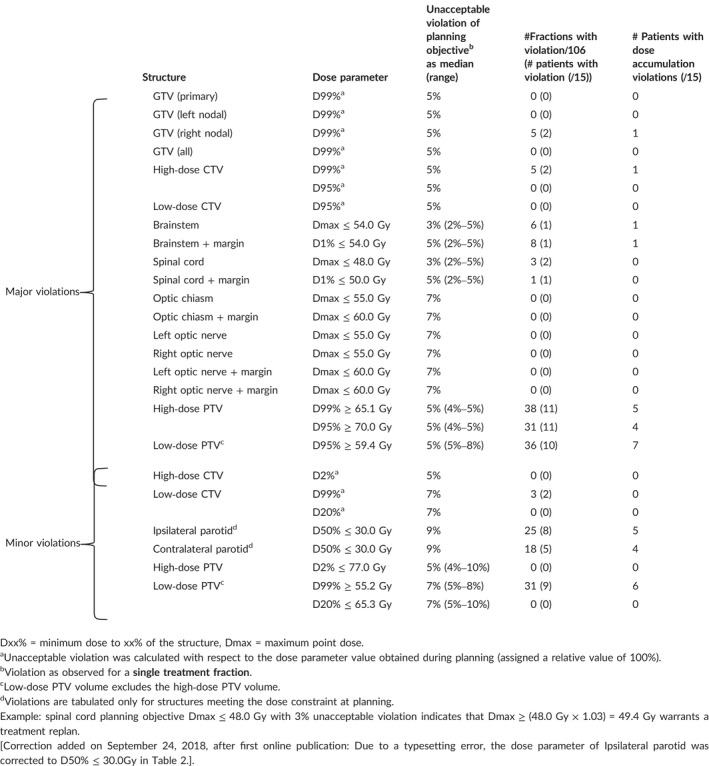
Priority structures, dosimetric parameters, and corresponding unacceptable violations indicating when a treatment replan is required, identified via the survey circulated to radiation oncologists specializing in head and neck cancer

### 3.B Protocol assessment

**Figure 1:**
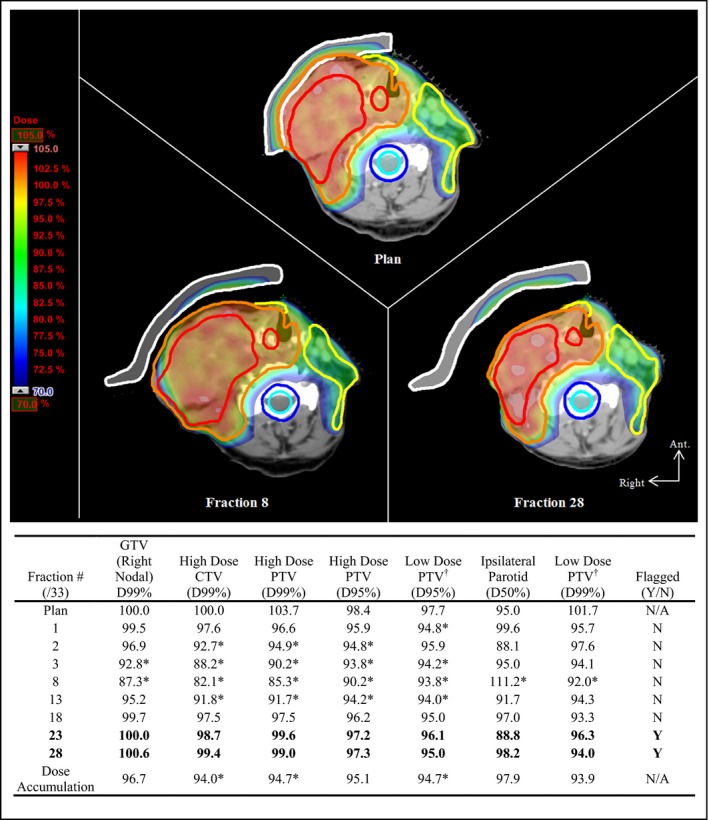
Localized change in body contour adjacent to the high‐dose PTV exhibited by Patient 1. Top: pretreatment CT and original plan with digitally rendered bolus (upper), synthetic CT image at fractions 8 (lower left) and 28 (lower right), and recalculated original plan with physical bolus placed on the external surface of the mask. 70% of the prescription dose (0.70 × 70 Gy = 49 Gy) approximately corresponds to the 3% major violation overdose of the spinal cord (49.4 Gy). Bottom: dose parameters calculated for major and minor violation criteria on fractions with CBCT acquisition. Entries are expressed as a percentage of planning objective or planned parameter value (see Table 2). **Bold** entries indicate fractions flagged by the protocol. (Contours: red — high‐dose GTV, orange — high‐dose PTV, yellow — low‐dose PTV, cyan — spinal cord, blue — spinal cord with margin, white — bolus). *Clinically significant deviation, according to the major/minor violation criteria (only those parameters violating Table 2 criteria are shown). ^†^Low‐dose PTV volume excludes the high‐dose PTV volume.

**Figure 2:**
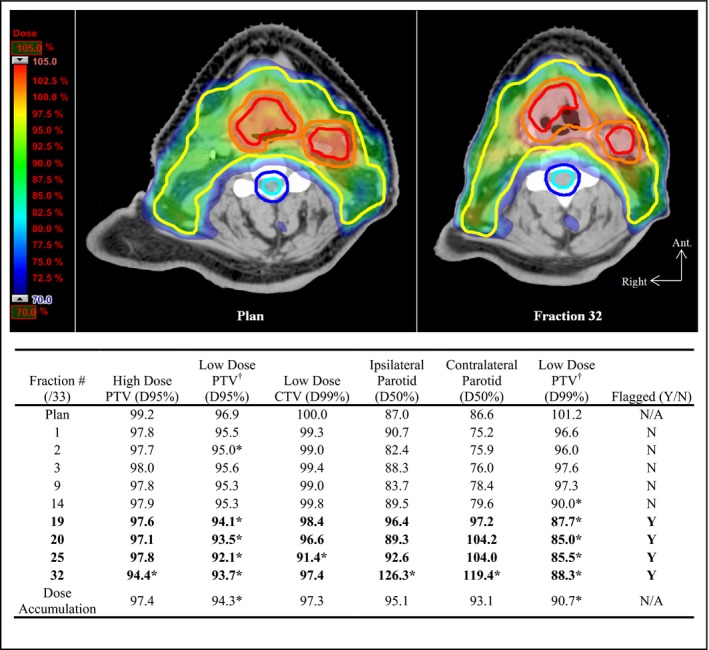
Change in body contour due to general weight loss effects exhibited by Patient 8. Top: pretreatment CT and original plan (left), CT image at fraction 32 and recalculated original plan (right). 70% of the prescription dose (0.70 × 70 Gy = 49 Gy) approximately corresponds to the 3% major violation overdose of the spinal cord (49.4 Gy). Bottom: dose parameters calculated for major and minor violation criteria on fractions with CBCT acquisition. Entries are expressed as a percentage of planning objective or planned parameter value (see Table 2). **Bold** entries indicate fractions flagged by the protocol. (Contours: red — high‐dose GTV, orange — high‐dose PTV, yellow — low‐dose PTV, cyan — spinal cord, blue — spinal cord with margin). *Clinically significant deviation according to the major/minor violation criteria (only those parameters violating Table 2 criteria are shown). ^†^Low‐dose PTV volume excludes the high‐dose PTV volume.

Figures 1 and 2 show examples of dramatic interfractional anatomy changes. In Fig. 1, the patient demonstrated localized changes in body contour adjacent to the high‐dose PTV. Major/minor violation criteria were exceeded as early as the first treatment fraction; however, the patient was not flagged by the protocol until fraction 23. Interestingly, this patient exhibited nonlinear trends in dosimetric violations: tumor growth and enlargement due to radiation‐induced edema increased until fraction 8 (high‐dose CTV volume increased by 25.6%) and corresponded to degradation in high‐dose target coverage. Subsequent tumor shrinkage of the high‐dose CTV to 59.8% of the original volume from fraction 8 through to the end of treatment led to a relative improvement of the target‐based violation parameters. Tumor growth caused a decrease in spinal cord Dmax from 47.9 Gy to 46.6 Gy, while shrinkage from fraction 8 onward corresponded to an increase to 49.3 Gy. While no violations were incurred for the spinal cord or spinal cord plus margin structures, the volume of the latter receiving the spinal cord violation dose (V49.4 Gy) decreased from 0.9% (plan) to 0.1% (fraction 8), increasing again to 1.3% (fraction 28). This suggests that isodose lines of the violation dose eventually began to encroach on the spinal cord. As expected, enlargement effects were more detrimental to tumor coverage than was subsequent shrinkage. Figure 2 shows changes in body contour as a result of weight loss. Here, flagging largely coincided with clinically significant changes in dose deposition, with respect to the violation criteria, and demonstrated more favorable protocol performance when compared with the former case.

Truth table results are presented in Table 3. Each fraction with CBCT acquisition is considered independently, giving 106 data points for the 15 patient cohort. For example, a fraction was classified as a true positive for target coverage (GTV, CTV, PTV) if the fraction was flagged by the institutional protocol and an unacceptable violation occurred in at least one of the GTV, high‐dose CTV, low‐dose CTV, high‐dose PTV, or low‐dose PTV dose parameters considered in Table 2. We assessed overall and target (GTV, CTV) performance with and without including PTV parameters: CTV to PTV margins preserve CTV coverage under setup uncertainties so that modest compromises in PTV coverage are expected. However, degradation of PTV D95% and D99% may indicate that high doses are consequently deposited in surrounding healthy tissue and motivated its selective inclusion. Notable among the truth table results are the low number of true‐positive values, that is, fractions flagged by the protocol with at least one parameter value exceeding the major violation criteria. By treating each fraction independently, the 5 of 15 patients imaged for the first three consecutive fractions may have slightly biased flagging performance toward that of early treatment fractions. For this demonstration, we were interested in quantifying the correct flagging result regardless of fraction number and in keeping with institutional imaging frequencies. Centers following the proposed assessment framework may elect to weigh flagging accuracy more heavily during the first half of treatment, when correctly identifying a patient requiring a replan may correspond to a greater cumulative dosimetric improvement.

**Table 3:** Results of the truth table analysis for major violation parameters expressed as # Fractions/106 (% of total fractions)

Structures	True positive (TP)	False negative (FN)	False positive (FP)	True negative (TN)
All major violations	18 (17%)	51 (48%)	8 (8%)	29 (27%)
All major violations Excluding PTV	0 (0%)	18 (17%)	26 (25%)	62 (58%)
Organs at risk (Brainstem, spinal cord, optics)	0 (0%)	10 (10%)	25 (24%)	70 (66%)
Target coverage (GTV, CTV, PTV)	18 (17%)	46 (43%)	8 (8%)	34 (32%)
Target coverage (GTV, CTV)	0 (0%)	8 (8%)	26 (24%)	72 (68%)

True positive: fraction (Fx) flagged, clinically significant deviation (CSD) in at least one parameter. False negative: Fx unflagged, CSD in at least one parameter. False positive: Fx flagged, no CSD. True negative: Fx unflagged, no CSD.

**Figure 3:**
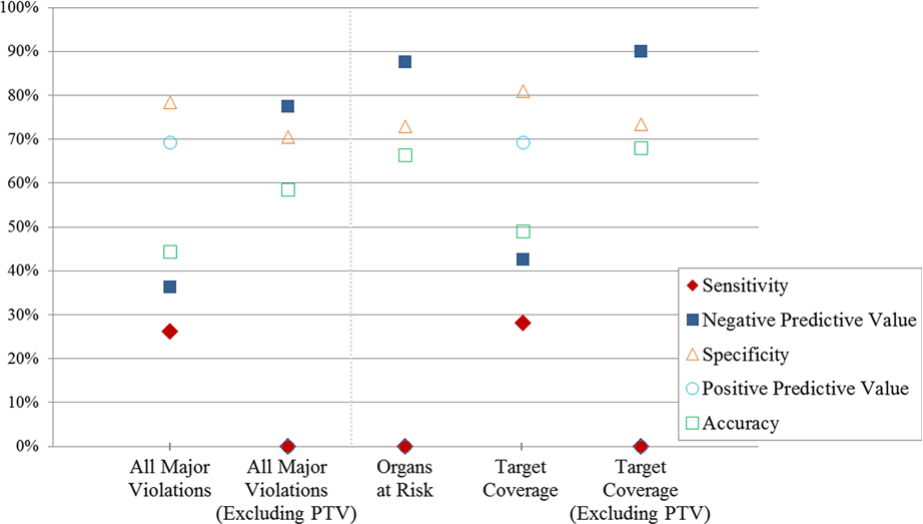
Efficacy of the 1.5 cm change in external body‐contour replan flag as a metric for identifying patients with clinically significant deviations in major violation parameters. (Sensitivity = TP/(TP + FN), Negative Predictive Value = TN/(TN + FN), Specificity = TN/(TN + FP), Positive Predictive Value = TP/(TP + FP), Accuracy = (TP + TN)/(TP + FN + FP + TN). See Table 3).

Figure 3 shows the sensitivity of the protocol, among other performance measures, in identifying treatment fractions with at least one parameter exceeding major violation criteria. A meaningful replan flag would have had high sensitivity (exceeding 80% 15 with 90% as a goal, for example) so that patients in need of a replanned treatment would be identified even at the expense of a moderate increase in the proportion of false positives, that is, trading gains in sensitivity for loss of specificity. In contrast, randomly flagging 20% of patient fractions under the assumption that 15% had clinically significant effects (similar to the relative number of flagged fractions and number of fractions exceeding the major violation criteria in the study cohort) yields a sensitivity of 27% (Appendix A). Given that the sensitivity of the current protocol (0%–28%) was comparable or poorer than that of a random flag, it is recommended that alternative flagging metrics be investigated; possible alternatives include change in face diameter, change in primary tumor volume, or a combination. Fundamental discrepancies in flag properties and RO‐based criteria, as further examined in the Discussion, are not expected to be efficiently overcome by simply adjusting the flagging threshold, for example, from 1.5 to 1.0 cm, to achieve various values for sensitivity on the receiver operating curve.

Negative predictive value and other parameters of secondary importance averaged ~60% for the >1.5 cm change in body contour‐based protocol, across all categories (all major violations, OAR, target coverage, etc.). Minor violation sensitivity averaged 44% across all categories, with all other parameters averaging 70% (results not shown). While metric performance was better for minor violations, sensitivity was still not high enough for the metric to be clinically relevant.

## 4 DISCUSSION

The practice of flagging patients exhibiting a change in external body contour exceeding 1.5 cm for possible reimmobilization, re‐CT, and/or replan was motivated by three‐dimensional (3D)‐conformal radiation therapy (3D‐CRT) and IMRT dosimetric calculation techniques. For example, a 1.5 cm isotropic decrease in the radius of a cylindrical volume with initial diameter of 15 cm corresponds to a ~5% increase in 6MV central axis dose. However, in general, target coverage decreased with progression throughout treatment as a result of geometric shifts of the GTV. Geometric shifts of high‐dose volumes relative to image‐guided radiation therapy alignment of bony anatomy are expected to be of general interest for ART protocol design. Due to the limited contrast of CBCT images, use of rigid bony alignment as standard of practice, and morphological (nonrigid) anatomical changes corresponding to geometric shifts in soft tissue structures relative to bony anatomy, replanning appears to be the most effective means for correcting the coincidence of high‐dose and CTV volumes. For the patient in Fig. 2, for example, an anterior shift of the GTV volume and posterior shift of the delivered high‐dose region corresponded to a net degradation in high‐dose PTV D95%, despite a local increase in deposited dose. A similar effect was observed in the inferior low‐dose PTV of patients with weight loss and consequent setup uncertainties about the neck and shoulders. Dose coverage was reduced as a result of the noncoincidence of target volumes which dominated the effect of increasing dose hot spots. Consequently, poor protocol performance was due to the incongruity between the dose‐based flagging practice and the geometrical nature of the major violation parameters. Further inconsistency was observed during protocol implementation where consult notes indicated that 10/15 patients were flagged due to erroneous shoulder positioning. Three further patients were flagged too late in treatment for an intervention/replan to result in a significant improvement in overall treatment quality. In all cases, no significant deviations in dose deposition affecting overall treatment quality (e.g., judged to be >5% across all 33 treatment fractions) were noted by treating clinicians and no replans were conducted. Dose accumulations calculated in this retrospective study were not available to clinicians at the time when replan decisions were made. Based on the dose accumulation data (not shown), replanning 12/15 patients may have improved all dose accumulation major violations; improving all dose accumulation major and minor violations would require the replanning of 13/15 of these “high‐risk” patients. However, the extent to which dose discrepancies can be improved through replanning depends on factors such as patient anatomy and number of fractions remaining. While inferring the timing and frequency of replans required to avoid violations falls outside the scope of our retrospective study, the literature suggests that 2–3 replans in the first half of treatment is the most effective.^11^, ^16^, ^17^

This study is limited by the necessary use of major/minor violation replanning criteria founded on physician experience and judgment rather than that of a formal quantitative analysis, as ART QUANTEC‐type guidelines do not yet exist. Stoll et al.^18^ have shown that even minor modifications of violation criteria result in significant changes in protocol performance in image‐guided radiation therapy correction strategies. Moderate departures from treatment planning practices, violation criteria, or implementation (e.g., automated flagging of body contour changes) in the present work are not expected to overcome the fundamental discrepancies in protocol capability, intention, and implementation. The limitations of deformable image registration are well known^19^ and exacerbated by the limited field of view of the CBCT images, CBCT artifacts, and extent of the anatomical changes observed. Anatomical effects may be underestimated as sections of the region of interest are “stitched” to the original CTsim. Small cohort size may limit the generalizability of results when inferring protocol performance for a diverse patient cohort. In addition, we expect the manual measurements of body contour change to be susceptible to uncertainty, especially about the 1.5 cm threshold. The scope of this study is to assess the impact of aggregate uncertainties via an end‐to‐end ART protocol assessment and so manual therapist measurements were retained in keeping with the institutional ART workflow. However, the assessment framework may be applied by centers specifically for prospective ART metric selection; once a center‐specific candidate ART protocol is identified, quantifications of implementation uncertainty may be assessed via repeat application of the proposed framework or *post hoc* analysis.

The literature suggests alternate metrics which may also be assessed via a similar truth‐table approach. Interfractional variation in face and neck diameter has been correlated with an increase in xerostomia scores^10^ and deviations in target coverage and hot spot location.^6^ Superior shifts in shoulder position have been shown to decrease low‐dose PTV coverage, with inferior shifts increasing dose to the brachial plexus.^20^ Despite the limited use of weight loss‐based metrics,^13^, ^16^ changes in patient weight correlate with an increase in spinal cord Dmax^6^, ^9^ and a medial shift of parotid centers of mass toward high‐dose regions.^7^, ^11^ A 1 cm change in body contour metric has been used by Brown et al.^12^ as a preliminary step in prospective ART cohort acquisition. The efficacy of the latter metric compared to RO decision‐making is not explicit in the study, which examines subsequent model development on the high‐risk cohort. In contrast, logistic regression and nomography have been used to assess the predictive capability of complex, multiparameter ART protocols which use data acquired prior to and during treatment.^12^, ^15^ Our proposed framework may be used to assess the predictive capabilities of simple standard of care or multimetric flags characterized by a normal/abnormal threshold.

## 5 CONCLUSION

A framework to quantify ART protocol performance in comparison to RO‐specified unacceptable dose violation criteria was demonstrated for a common ART flagging metric: >1.5 cm change in external body contour. This framework successfully identified a mismatch between the flag's intended purpose of identifying changes in central‐axis dose and the physician priorities to correct for geometric shifts. This work suggests that centers may similarly benefit by quantifying ART performance according to center‐specific requirements.

## CONFLICT OF INTEREST

The authors declare no conflict of interest.

## APPENDIX A

### A.1

(The following uses the notation TP = true positive, FP = false positive, TN = true negative, FN = false negative in keeping with Table 3 and Fig. 3.)

To elaborate on how the 27% sensitivity of a random flag on a comparable patient sample is derived, we first assumed that approximately 26/121 fractions are flagged (TP + FP = 20%) while only 15% of fractions exhibit a clinically significant effect (TP + FN = 15%). These proportions are in keeping with that of the study cohort. In addition, TP + FN + FP + TN = 100%. Furthermore, from analysis of receiver operating characteristic curves, we assume that a *random* flag is such that sensitivity is approximately equal to (1 − specificity):

$$Sensitivity=1-Specificity=1-\frac{\mathrm{TN}}{TN+FP}$$

⟹TP×(TN+FP)=FP×(TP+FN)

⟹0.85×TP=0.15×FP

⟹TP=0.18×FP

And so, TP + FP = 20% implies that FP = 16%–17%. The truth table can then be completed by calculating TP, FN, TN as follows:

**Table:**

	Clinically significant effects	No clinically significant effects	
Flagged	TP = 4%	FP = 16%	20%
Unflagged	FN = 11%	TN = 69%	80%
	15%	85%	

i.e., sensitivity = 4%/(4% + 11%) = 27%.
